# Accuracy of Mobile Device–Compatible 3D Scanners for Facial Digitization: Systematic Review and Meta-Analysis

**DOI:** 10.2196/22228

**Published:** 2020-10-23

**Authors:** Hang-Nga Mai, Du-Hyeong Lee

**Affiliations:** 1 Institute for Translational Research in Dentistry Kyungpook National University Daegu Republic of Korea; 2 Department of Prosthodontics, School of Dentistry Kyungpook National University Daegu Republic of Korea

**Keywords:** accuracy, facial digitization, facial scanners, systematic review, meta-analysis

## Abstract

**Background:**

The accurate assessment and acquisition of facial anatomical information significantly contributes to enhancing the reliability of treatments in dental and medical fields, and has applications in fields such as craniomaxillofacial surgery, orthodontics, prosthodontics, orthopedics, and forensic medicine. Mobile device–compatible 3D facial scanners have been reported to be an effective tool for clinical use, but the accuracy of digital facial impressions obtained with the scanners has not been explored.

**Objective:**

We aimed to review comparisons of the accuracy of mobile device–compatible face scanners for facial digitization with that of systems for professional 3D facial scanning.

**Methods:**

Individual search strategies were employed in PubMed (MEDLINE), Scopus, Science Direct, and Cochrane Library databases to search for articles published up to May 27, 2020. Peer-reviewed journal articles evaluating the accuracy of 3D facial models generated by mobile device–compatible face scanners were included. Cohen d effect size estimates and confidence intervals of standardized mean difference (SMD) data sets were used for meta-analysis.

**Results:**

By automatic database searching, 3942 articles were identified, of which 11 articles were considered eligible for narrative review, with 6 studies included in the meta-analysis. Overall, the accuracy of face models obtained using mobile device–compatible face scanners was significantly lower than that of face models obtained using professional 3D facial scanners (SMD 3.96 mm, 95% CI 2.81-5.10 mm; z=6.78; *P*<.001). The difference between face scanning when performed on inanimate facial models was significantly higher (SMD 10.53 mm, 95% CI 6.29-14.77 mm) than that when performed on living participants (SMD 2.58 mm, 95% CI 1.70-3.47 mm, *P*<.001, df=12.94).

**Conclusions:**

Overall, mobile device–compatible face scanners did not perform as well as professional scanning systems in 3D facial acquisition, but the deviations were within the clinically acceptable range of <1.5 mm. Significant differences between results when 3D facial scans were performed on inanimate facial objects and when performed on the faces of living participants were found; thus, caution should be exercised when interpreting results from studies conducted on inanimate objects.

## Introduction

Oral and facial rehabilitation involves comprehensive diagnosis and treatment planning [1,2]. Facial morphology assessment is vital for the diagnosis of maxillofacial anomalies, surgery, fabrication of prostheses, and postoperative evaluation [2,3]. Esthetics and prognosis of treatment outcomes can be improved through simulation performed on the 3D facial models of patients [4]. The conventional method for generating facial models of patients is physical facial impression, in which a replica of the face is fabricated using elastomeric materials and a gypsum cast [5,6]. However, the method is uncomfortable for patients because their face is covered with materials during the impression-taking process [6]. In addition, the dimensional accuracy of the physical facial impression model is affected by several factors, including the viscosity of the impression materials, setting time, storage conditions, and time interval from material mixing to stone pouring of the casts [7,8]. Furthermore, the human face is made up of complex anatomical structures with complicated skin textures and colors, which makes realistic replication of the face challenging.

Modern digital technologies have revolutionized the facial impression method by enabling 3D facial morphology to be captured using noncontact optical facial scanning devices [9,10]. Digital impression does not require conventional laboratory work or the use of impression materials, thus reducing the discomfort and chair time of the patients. Compared with facial stone casts, wherein only direct anthropometric measurements of the faces can be performed for facial analyses, virtually reconstructed models of the face can be utilized for multidisciplinary purposes [11-13]. Facial landmarks can easily be extracted from a digital facial model, and the digitized data format enables image merging and advanced dimensional analyses, such as surface-to-surface distance measurements and volume misfit evaluations, using analytical computer software [3,14-17]. In addition, digital facial scanning provides an efficient basis for dental education and facial recognition [18-20].

Stationary facial scanning systems based on stereophotogrammetry technology were first introduced in dentistry [21]. However, because of the encumbrance and high cost of this technology, handheld scanning systems using laser or structured-light technology were developed [21-23]. Although most professional handheld scanners are considered acceptable in terms of their scan image quality, they are expensive and often require considerable training time to learn their complex scanning protocols [3,24,25]. Alternatively, 3D sensor cameras based on structured-light technology have been developed for smartphone and tablet devices [15,26-28]. An advantage of using mobile devices for face scanning is their user-friendly operation; this reduces the training time for users [15,29]. Apps can be developed and customized for specific purposes by using open source scripts and software coding [15,29]. Moreover, when an external attachment-type 3D sensor camera is used, the position of the camera is controllable in the mobile-device system [27,29].

Facial scanning using a mobile device 3D sensor camera has been attracting a lot of interest in recent years because it is highly portable and cost-effective and because of the popularity of mobile devices [29]. Smartphone- and tablet-compatible 3D facial scanners have been reported to be an effective tool for clinical use in prosthodontic treatment [27,30-33]. However, the accuracy of the digital facial impression obtained with mobile device–compatible face scanners has not been explored. The purpose of this systematic review and meta-analysis was to investigate the accuracy of mobile device–compatible face scanners for facial digitization.

## Methods

### Study Design

This study was designed based on PRISMA guidelines (Preferred Reporting Items For Systematic Reviews and Meta-Analyses) [34]. This review was not preregistered on PROSPERO. Accuracy was defined as a dimensional discrepancy between the digital facial impression made by a mobile device–compatible face scanning camera and reference image data set. The PICO (population, intervention, comparison, and outcomes) question was as follows: Are digital facial impressions (population) obtained with mobile device–compatible 3D facial scanning cameras (intervention) equivalent to those of professional handheld face scanners (comparison) in terms of accuracy (outcomes)?

### Search Strategy

Peer-reviewed studies published until May 27, 2020 were searched using the following formulated Boolean operator: (*digital facial impression* OR *3D virtual face* OR *digital face*) AND (*optical scanner* OR *3D scanner* OR *stereophotogrammetry* OR *structured light* OR *laser scanner* OR *depth sensor cameras* OR *depth-sensing cameras*) AND (*smart device* OR *mobile* OR *smartphone* OR *tablet* OR *notebook* OR *laptop*) AND (*validation* OR *comparison* OR *accuracy* OR *agreement* OR *reliability* OR *precision* OR *reproducibility*). The Boolean operator was applied in major electronic databases including PubMed (MEDLINE), Scopus, Science Direct, and Cochrane Library. The Google Scholar search engine was used to find additional articles by combining the related MeSH (Medical Subject Headings) terms and text words. No automatic limiter setting was used during the searches to prevent unwanted filtering of related articles. EndNote software (version 9.2, Clarivate Analytics Inc) was used to manage the articles’ references.

### Inclusion and Exclusion Criteria

Inclusion and exclusion criteria were set based on the study design, objectives, interventions, and measurement results. The search was limited to articles published in English only. The inclusion criteria for meta-analysis were low risk of bias, low concern for applicability, and relevant numeric data for pool-weighted estimation using the Cohen *d* statistical method. Accordingly, randomized and nonrandomized controlled trials, cohort studies, case-control studies, and cross-sectional studies that were performed with human participants and on inanimate objects, reporting quantitative assessments of digital facial models obtained with 3D facial scanners and mobile device–compatible 3D facial scan cameras were included in this review. Conversely, conference papers, case reports, case letters, epidemiologic studies, and author or editorial opinion articles were excluded. Original studies that used only 2D images or did not include mobile device–compatible 3D facial scanners were not reviewed, and studies in which the accuracy could not be quantitatively determined were not considered for analysis.

### Data Collection

Two reviewers (H-NM and D-HL) independently participated in collecting, screening, and selecting the potential studies based on the information provided by the titles and abstracts. The full texts of relevant articles were assessed and reviewed by both reviewers. The papers that satisfied all the inclusion criteria were considered eligible for review. The following information was collected from full-text papers and recorded on an electronic spreadsheet (Office Excel, Microsoft Inc): authors, year of publication, study purpose, participant information (sample size, mean age, age range, and gender proportion), scanning methods (scanning device, capture technology, working condition, and scanning process), reference standard for validation (direct anthropometry or another 3D scanning device), types of measurement performed (linear distances or surface-to-surface deviation), number of measurements (number of landmarks, measurement times, and raters), measurement results (mean, estimation errors, and types of statistical analysis), and major conclusions. Articles with missing data or unreliable data were excluded from the meta-analysis. The agreement (κ) between the 2 reviewers was calculated. In case of disagreement, a discussion between the 2 reviewers was conducted to resolve the issues.

### Quality Assessment and Meta-Analysis

The risk of bias and concern for applicability based on 4 bias domains—patient selection, index test, reference standard, and flow and timing—were assessed by the 2 reviewers using the Quality Assessment Tool for Diagnostic Accuracy Studies-2 (QUADAS-2) [35].

The random- or fixed-effects model was used to analyze the standardized mean difference (SMD) between the experimental and reference data sets to investigate the effect size estimate and the confidence intervals of SMDs using Cohen *d* [36]. Heterogeneity was evaluated using the Cochran *Q* test based on the Higgins *I*^2^ statistic [37], where a higher *I*^2^ value indicated a stronger heterogeneity. When the *Q* test indicated high heterogeneity across studies (*P*<.05) or *I*^2^>50%, the random-effects model was selected, and subgroup analysis was performed [38]. The subgroup was defined based on the participants or inanimate objects investigated.

Publication bias was assessed using the Egger linear regression statistical test and visually inspected using funnel plots. Meta-analyses were performed using the meta package for R software (version 3.6.0, R Foundation for Statistical Computing Platform); the significance level was set at .05. The robvis package (version 0.3.0) was used to visualize the risk-of-bias assessment results [39].

## Results

### Search Results

**Figure 1:**
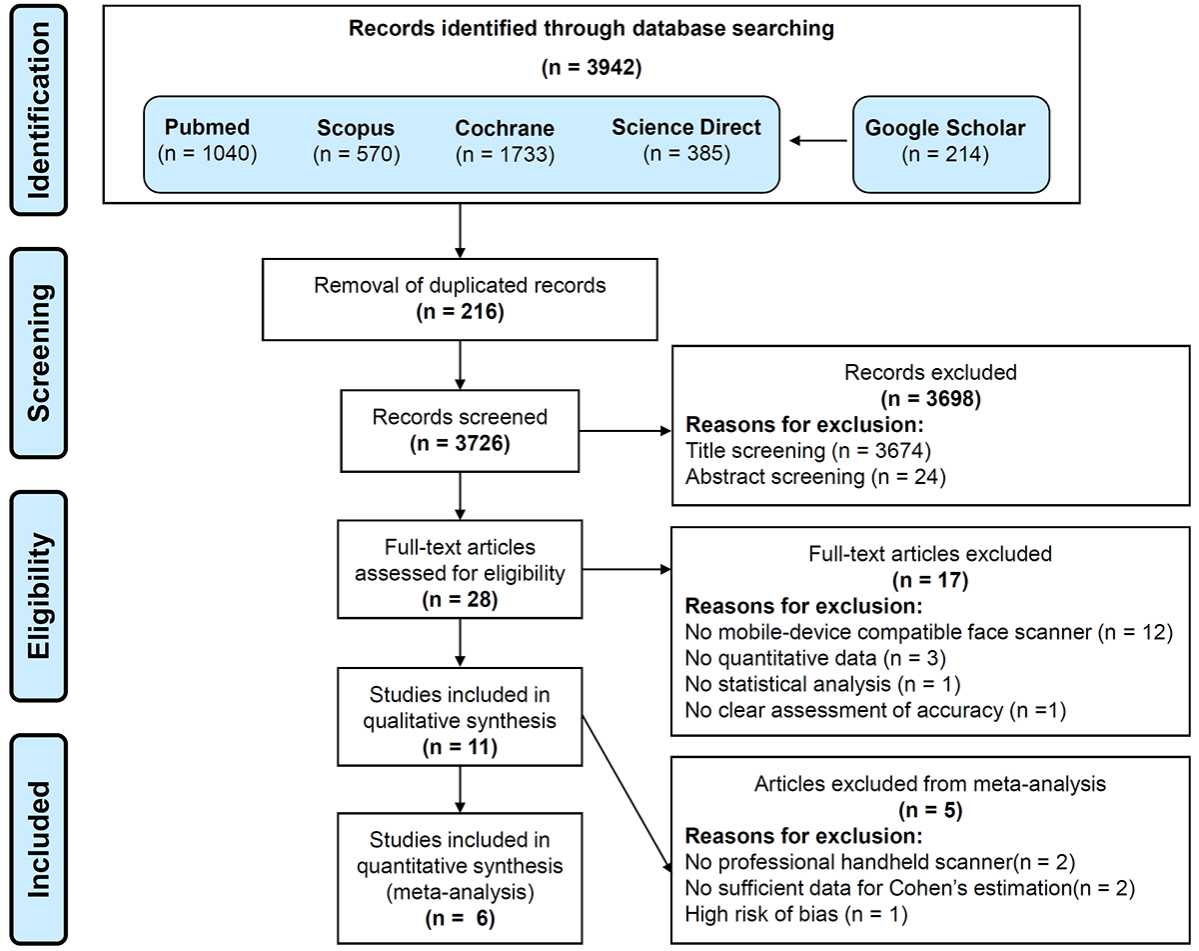
PRISMA flow diagram summarizing search strategy and search results.

The search resulted in a total of 3942 articles, which were reduced to 3726 articles after removing 216 duplicates. In the title screening process, 3674 articles that were outside the scope of this review were excluded, thereby leaving 52 articles for abstract screening. After the exclusion of 24 articles with irrelevant abstracts, the full texts of 28 articles were read and assessed, and 11 articles were considered eligible for this review. Of these, 6 articles were included in the global meta-analysis, 4 articles were included in the living person face subgroup analysis, and 3 articles in the inanimate face subgroup analysis. The results of the searching and screening process are summarized in Figure 1. There was substantial interrater agreement (κ=0.90).

### Quality Assessment and Applicability Concerns

**Figure 2:**
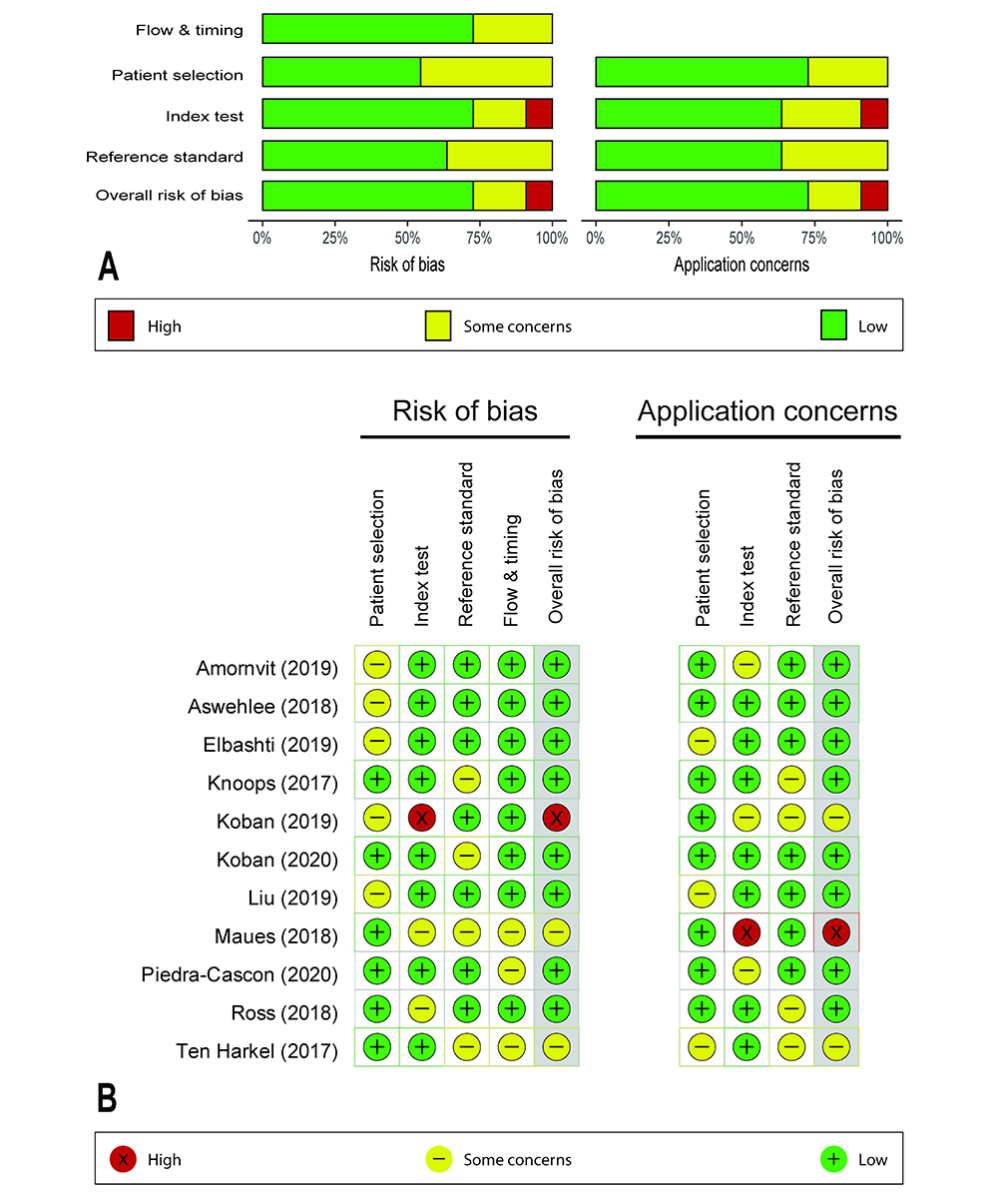
Quality assessment results according to Quality Assessment Tool for Diagnostic Accuracy Studies-2 guidelines.

The quality assessment results from the Quality Assessment Tool for Diagnostic Accuracy Studies-2 showed that among the 11 studies included, one study [40] had a high risk of bias, and another study [41] had a high concern for applicability (Figure 2). There were 2 studies [41,42] showing some risk of bias, and there were 2 studies [40,42] for which there were some concerns for applicability. The patient selection and index test had a higher risk of bias than those of other domains in some studies because of unclear statements regarding the methods employed for random sampling [28,43] or the small number of participants included [5,15]. For applicability, the major concerns arose in the index test domain because several studies did not describe the scanning procedures in detail or did not provide sufficient information about the scanning devices [27,28,40,41].

### Study Characteristics

Extracted data were organized according to the characteristics of the studies (Table 1). The characteristics of the mobile device–compatible face scanners that were investigated are summarized in Multimedia Appendix 1.

**Table 1:** Characteristics of the included studies.

Study	Participant or specimen	Mobile device, face scanner	Reference	Landmark, n	Measurement	Major findings
Amornvit (2019)[28]	1 mannequin head	iPhone X (Apple Inc), FaceApp (Bellus3D Inc)	Manual measurement	N/A^a^	Δ*x*, Δ*y*, and Δ*z*	Δ*x*: range 10-50 mm; Δ*y*: range 50-120 mm; failure to record the details in the *z*-axis
Aswehlee (2018)[5]	1 impression cast	Scanify (Fuel 3D Technologies Ltd)	CT^b^	3D point clouds	RMSE^c^	The most accurate noncontact 3D digitizer for maxillofacial defects was Vivid 910 (Minolta Corp), followed by Danae (NEC Engineering), 3dMD (3dMD LLC), and Scanify (*P*<.001).
Elbashti (2019)[15]	1 impression cast	iPhone 6 (Apple Inc), 123D Catch App (Autodesk Inc)	CT	3D point clouds	RMSE	Smartphone 3D modeling was not as accurate as that of the commercially available laser scanning, with higher RMSE values in the defect area representing the depth of the defect.
Knoops (2017)[29]	8 (4 male, 4 female)	Structure Sensor (Occipital Inc)	SP^d^	3D point clouds; 4	RMSE	RMSE of the Structure Sensor was significantly higher than that of M4D Scan (Rodin4D) (*P*=.008). Structure Sensor lacks hardware and software to accurately characterize areas with complex shape and high curvature but is good at describing general facial forms.
Koban (2019)[40]	4 cadaver heads (N/A)	Sense (3D Systems Inc); iSense (3D Systems Inc)	N/A	3D point clouds	RMSE	Artec Eva (Artec Group) provided significantly more accurate results than those of the Sense (*P*<.001) and the iSense devices (*P*<.001). The Sense was more accurate than the iSense scanner; however, the difference was not significant (*P*=.12).
Koban (2020)[44]	30 (15 male, 15 female), 1 mannequin head	Sense (3D Systems Inc)	SP	3D point clouds	RMSE	Whole face <1.0 mm (RMSE 0.516, SD 0.109 mm).
Liu (2019)[43]	2 impression cast (male)	Scanify (Fuel 3D Technologies Ltd)	CT	13	11 linear deviations (Δ*x*, Δ*y*, and Δ*z*)	Overall, linear deviations <1 mm for Scanify. The mean overall difference <0.3 mm between Scanify (mean 0.74, SD 0.089 mm) and Vectra (mean 0.15, SD 0.015 mm) images.
Maues (2018)[41]	10 (5 male, 5 female)	Kinect (Microsoft Inc)	SP	10	7 linear distances (mean difference)	Mean difference between scanning methods was 0.3 (SD 2.03 mm), showing reasonable accuracy. The mean difference between the images taken with Kinect) was 0.1 (SD 0.6 mm; *P*<.05) showing good accuracy. Kinect appears to be an interesting and promising resource for facial analysis.
Piedra-Cascón (2020)[27]	10 (2 male, 8 female)	Face Camera Pro (Bellus3D Inc)	Manual measurement	6	RMSE	Face Camera Pro exhibited a trueness RMSE of 0.91 mm and a precision RMSE of 0.32 mm.
Ross (2018)[45]	16 (8 male, 8 female)	iPhone 7 (Apple Inc), Camera+ app (tap tap tap LLC); RealSense (Intel Corp)	Structured light	3D point clouds	RMSE	No significant differences in RMSE values between iPhone scans with 90 photographs (RMSE 1.4, SD 0.6 mm), 60 photographs (RMSE 1.2, SD 0.2 mm), or 30 photographs (RMSE 1.2, SD 0.3 mm). RealSense had significantly higher RMSE than the iPhone experimental groups (*P*<.001).
Ten Harkel (2017)[42]	34 (10 male, 24 female)	RealSense (Intel Corp)	SP	3D point clouds	RMSE	RealSense depth accuracy was not affected by facial palsy (RMSE 1.48, SD 0.28 mm) compared to a healthy face (RMSE 1.46, SD 0.26 mm) or Sunnybrook poses^e^ (*P*=.76). However, distance of the patients to the RealSense device was shown to affect accuracy, where the highest depth accuracy (1.07 mm) was measured at a distance of 35 cm.

^a^N/A: not applicable.

^b^CT: computed tomography.

^c^RMSE: root-mean-square error (surface-to-surface).

^d^SP: stereophotogrammetry.

^e^Sunnybrook poses are a facial grading system for evaluating facial movement outcomes, both at rest and through 5 facial expressions based on voluntary movements (forehead wrinkle, gentle eye closure, open mouth smile, snarl, and lip pucker) [42].

Among the 11 studies included, 6 were conducted on adult volunteers or patients [27,29,41,42,44,45] with a mean age of 35.50 years (SD 8.50; range 24-59). The number of participants in these studies ranged from 8 to 34, with 2 to 15 male and 4 to 15 female participants. The other 5 studies were conducted using inanimate objects such as impression casts of the face [5,15,43] or mannequin heads [28,44], and 1 study [40] was conducted on human cadaver heads. Stereophotogrammetry [29,41,42,44], computed tomography [5,15,43], and high-resolution structured-light handheld scanning [40,45] were used as the reference measurements for comparison, and 2 studies [27,28] used manual interlandmark distance as the reference measurement.

For the evaluation, most studies [5,15,27,29,40-42,44,45] measured the global surface-to-surface deviation between the reference and test images by calculating the root-mean-square error (RMSE) of the superimposed 3D images using analytical computer software, with a higher RMSE value indicating a higher surface deviation; however, 3 studies [28,41,43] compared the distances between facial landmarks on a digitized face with those between respective landmarks on a physical model obtained using the manual measurement method. Among them, 1 study [41] evaluated both the global surface-to-surface deviation and interlandmark linear distances, and the deviation was assessed along the *x*-axis (horizontal length), *y*-axis (vertical length), and *z*-axis (depth) in another study [28].

### Meta-Analysis

**Figure 3:**
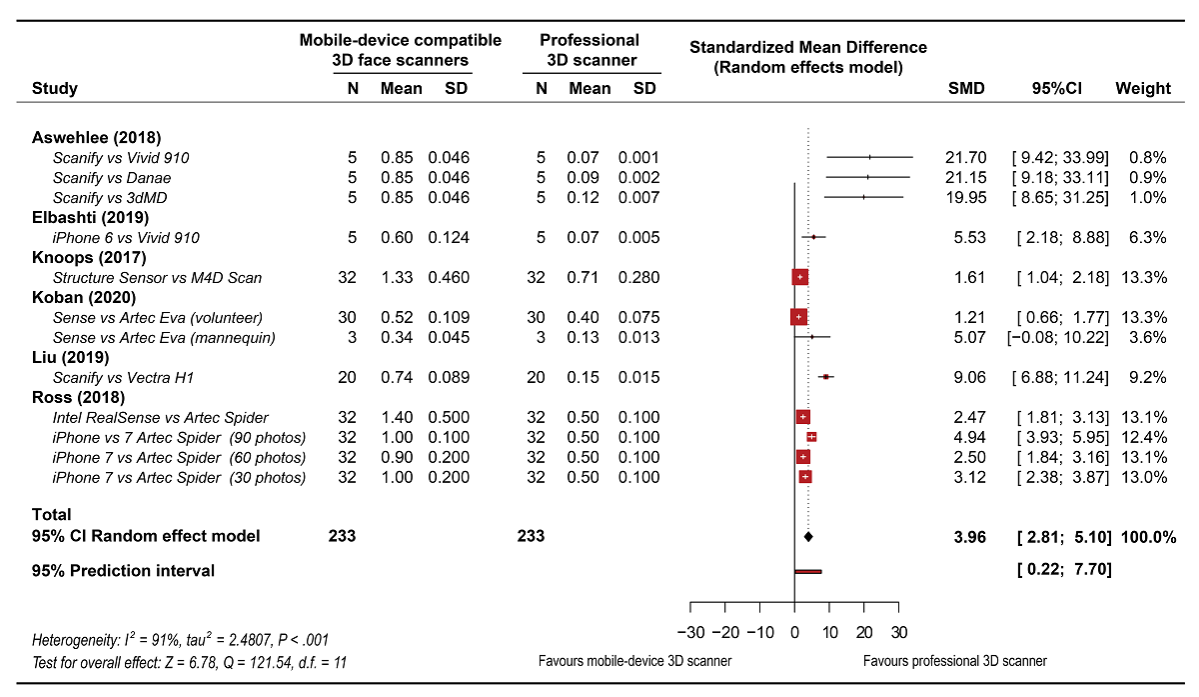
Global meta-analysis results of comparison of facial models obtained using mobile device–compatible face scanners versus professional face scanners.

**Figure 4:**
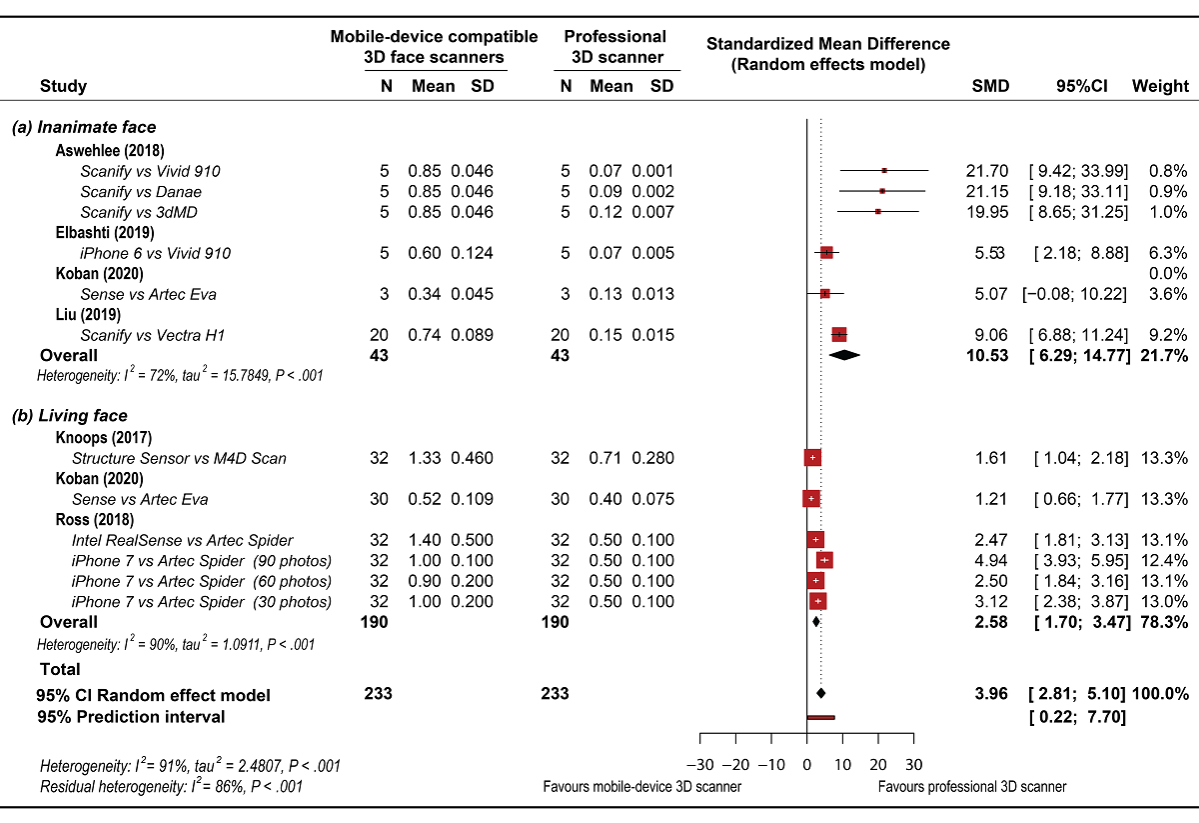
Subgroup meta-analysis results of comparison of facial models obtained using mobile device–compatible face scanners versus professional face scanners. (a) 3D facial scans performed on inanimate objects, (b) 3D facial scans performed on living persons.

The global analysis revealed heterogeneity (*I*^2^=91% *P*<.001). Random-effects models were selected for both global and subgroup meta-analyses based on the heterogeneity among the studies. In general, the accuracy of facial models obtained with mobile device–compatible face scanners was significantly lower than that of facial models obtained using professional face scanners (SMD 3.96 mm, 95% CI 2.81-5.10 mm, *z*=6.78, *P*<.001; Figure 3). Results from the subgroup analysis revealed a significant difference between the subgroups (Figure 4). The difference between the mobile device–compatible and professional face scanners was significantly higher for the face scans of inanimate facial objects (SMD 10.53 mm, 95% CI 6.29-14.77 mm) than for those of living participants (SMD 2.58 mm, 95% CI 1.70-3.47 mm, *P*<.001, *df*=12.94).

**Figure 5:**
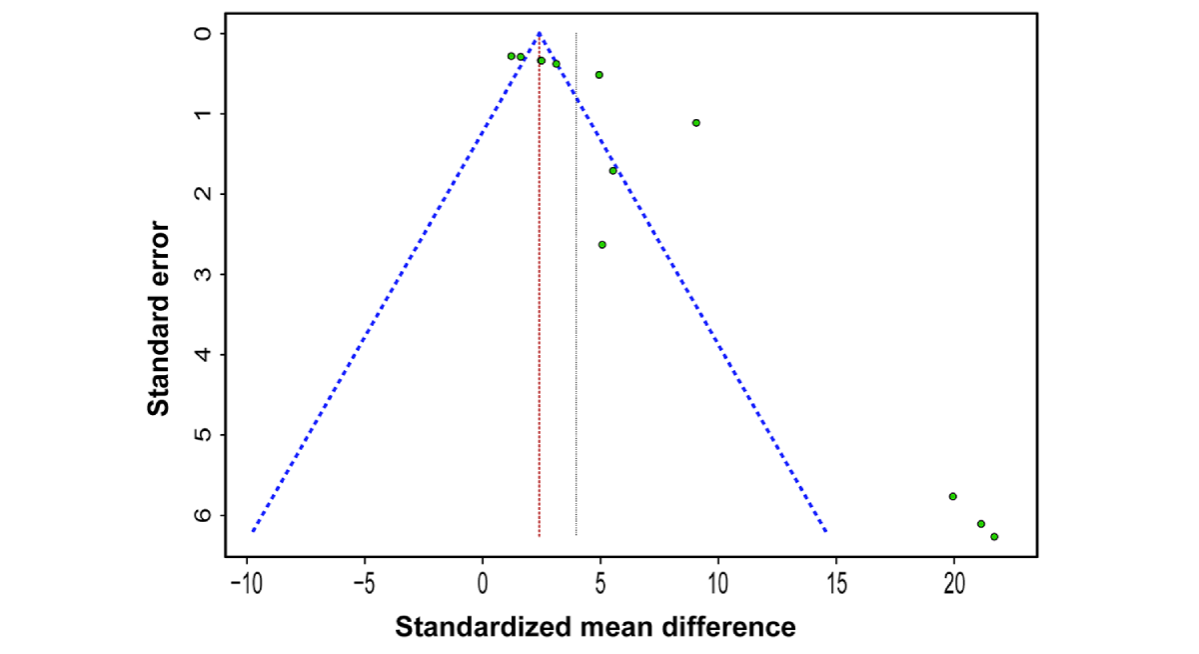
Funnel plot showing of publication bias assessment.

The funnel plot showed asymmetry arising from 3 distinct points with different effect estimates (Figure 5). Regarding publication bias, Egger test results showed an intercept of 3.9 (95% CI 1.94-5.86, *t*=3.792, *P*=.004).

## Discussion

### Principal Findings

We aimed to investigate the accuracy of mobile device–compatible face scanners in facial digitization. Mean discrepancy values of the digitized face obtained using mobile device–compatible 3D facial scanners ranged from 0.34 to 1.40 mm in articles included in this systematic review. The meta-analysis revealed that mobile device–compatible 3D facial scanners were less accurate than their professional 3D counterparts. The reliability of a digital face scanner can be classified into 4 categories: highly reliable (deviation <1.0 mm), reliable (deviation 1.0 mm-1.5 mm), moderately reliable (deviation 1.5 mm-2.0 mm), and unreliable (deviation >2.0 mm) [46]. For clinical application, deviations <1.5 mm were considered acceptable [3,47,48]. Based on the classifications, mobile device–compatible 3D facial scanners were considered acceptable for clinical use even though their accuracies were lower than those of the professional 3D facial scanners. Amornvit et al [28] and Liu et al [43] reported that mobile device–compatible face scanners are comparable to professional 3D facial scanners when scanning simple and flat areas of the face such as the forehead, cheeks, and chin. However, scanning accuracy was relatively low when mobile device–compatible face scanners were used to capture complex facial regions, such as the external ears, eyelids, nostril, and teeth [28,44,45]. Higher inaccuracy was found in the facial areas with defects, depending on the depth of the defect [15]. Thus, careful consideration in accordance with the purpose and the person might be needed when using mobile device–compatible face scanners.

In the preliminary stages, smartphone-based 3D scanners used a multiphotogrammetry approach that captured several photographs of the object from different views and matched common features in the photographs to establish a 3D model of the object by using dedicated smartphone software apps [15,45]. The resolution of a 3D image depended on the number of reconstructed polygons that were calculated by the software algorithm based on the resolution of the captured images [49]. The working principle is similar to that of professional stereophotogrammetry facial scanning systems; however, professional systems usually use digital single-lens reflex cameras that have higher pixel densities with better noise reduction software and higher ISO settings compared with those of smartphone cameras [50]. The accuracy of smartphone multiphotogrammetry in facial data acquisition was reported as 0.605 (SD 0.124) mm by Elbashti et al [15]. In another study by Ross et al [45], the mean discrepancy of scan data obtained using smartphones ranged from 0.9 mm to 1.0 mm, depending on the number of photographs taken during scanning. In recent years, infrared structured-light depth-sensing cameras have been incorporated in mobile devices to facilitate 3D optical scans [51]. 3D depth-sensing cameras work by the time-of-flight principle, measuring the time taken for light emitted by an illumination unit to travel to an object and back to the sensor array [52,53]. The 3D images are then reconstructed based on a depth map of the object and surroundings [54]. Although smartphone depth-sensing cameras share similar working principles with professional laser scanning systems, laser systems are more sensitive to depths because they are built with higher sensitivity sensors [15,23]. Amornvit et al [28] reported that the 3D depth-sensing sensor scanner of a smartphone is reliable in linear measurement at the frontal plane, but it has less accuracy in depth measurement compared with that of professional face scanners. Depth-sensing cameras can also be used separately and attached or plugged into smartphones, tablets, or laptop computers to acquire 3D scans [27,29,40-42,44]. Because the quality of facial scanning is also affected by the performance of compatible mobile devices when external depth-sensing cameras are used, the resulting accuracy might vary widely and should be evaluated for each combination of depth-sensing camera and mobile device.

Subgroup meta-analysis showed that the accuracy of 3D facial scans performed on living persons was significantly different compared with those performed on inanimate objects. This result implies that the outcomes of in vitro or laboratory studies could be different from those obtained from people. Thus, based on the findings of this review, we recommend using living persons for related research on mobile device–compatible face scanning. Caution should be exercised when scanning the orbital, nasolabial, and oral regions on the face of a living person to minimize the discrepancies caused by motion artifacts [16,24]. Subconscious nose breathing, eye blinking, and lip twitching should also be carefully considered as these are the main sources of involuntary facial movements [16]. Ozsoy et al [17] reported that changes in facial expressions could affect the reproducibility and reliability of a scan, with the highest error values observed for a frightened facial expression and the lowest value observed for neutral facial expression. To reduce motion artifacts, the person should be instructed to maintain a neutral facial expression and avoid any head movement during image acquisition [55]. Another concern is that human faces contain complex skin textures, pores, freckles, scars, and wrinkles. Some artifacts or missing scan data appear as holes, originating from surfaces that are difficult to capture, such as eyebrows, eyelashes, and hairlines [29]. Small empty holes can be repaired using image processing software that uses neighboring areas that are morphologically similar; however, large defects can cause difficulties in the stitching process because of the lack of reference [24]. In addition, human faces vary in shape and are not perfectly symmetric, thus may appear different in different angles of view [56]. This phenomenon might cause some artifacts when the multiphotogrammetry approach is used because the 3D model of an object is reconstructed by matching common facial features in the captured photographs.

A limitation of this review is that the review protocol was not preregistered on PROSPERO. Most included studies are not directly correlated with clinical treatment outcomes due to the difficulty of performing clinical studies to assess the accuracy of scanners. However, the findings of this review show great promise for the clinical use of mobile device–compatible face scanners. Another limitation of this systematic review is the small number of included studies. The limited number of studies show high heterogeneity and funnel plot asymmetry. Regarding publication bias, the Egger test result was significant (*P*=.004). Heterogeneity can cause funnel plot asymmetry when a correlation between intervention effects and study sizes is present [57]. Further examination was performed on the eligibility of a study that showed distinctly larger effect estimates [5], and we included the study [5] in the meta-analysis because it was conducted in an environment of a scanning intervention and was methodologically scientific. Although the inclusion of this study [5] increased the heterogeneity among studies and funnel plot asymmetry, the results were fundamentally attributed to a small number of articles [58]. All eligible papers included in the review were published between 2017 and 2020 due to the novelty of the research topic. A random-effects model is often used in meta-analyses for studies with heterogeneity. Random effects meta-analyses weigh studies more equally than fixed-effect analyses by incorporating the variance between studies [58]. Therefore, in this review, based on heterogeneity and funnel plot asymmetry, random-effects models were selected for global and subgroup analyses. Additional controlled in vitro and randomized clinical trials will be needed to reinforce the impact of review articles. Moreover, considering the rapid development of face scanning in the medical field, diverse investigations with newly developed devices and systems need to be continuously performed.

### Conclusions

Overall, the accuracy of mobile device–compatible face scanners in 3D facial acquisition was not comparable to that of professional optical scanning systems, but it was still within the clinically acceptable range of <1.5 mm in dimensional deviation. There were significant differences between 3D facial scans performed on inanimate objects and living persons; thus, caution should be exercised when interpreting the results from studies conducted on inanimate objects.

## Appendix

Multimedia Appendix 1 Commercial mobile device–compatible face scanners investigated in the studies.

## Abbreviations

RMSE: root-mean-square error
SMD: standardized mean difference

## References

[1] Spear FM, Kokich VG (2007). A multidisciplinary approach to esthetic dentistry. Dent Clin North Am.

[2] Jazayeri HE, Kang S, Masri RM, Kuhn L, Fahimipour F, Vanevenhoven R, Thompson G, Gheisarifar M, Tahriri M, Tayebi L (2018). Advancements in craniofacial prosthesis fabrication: A narrative review of holistic treatment. J Adv Prosthodont.

[3] Zhao YJ, Xiong YX, Wang Y (2017). Three-dimensional accuracy of facial scan for facial deformities in clinics: a new evaluation method for facial scanner accuracy. PLoS One.

[4] Bidra AS (2011). Three-dimensional esthetic analysis in treatment planning for implant-supported fixed prosthesis in the edentulous maxilla: review of the esthetics literature. J Esthet Restor Dent.

[5] Aswehlee AM, Elbashti ME, Hattori M, Sumita Y, Taniguchi H (2018). Feasibility and accuracy of noncontact three-dimensional digitizers for geometric facial defects: an in vitro comparison. Int J Prosthodont.

[6] Alsiyabi AS, Minsley GE (2006). Facial moulage fabrication using a two-stage poly (vinyl siloxane) impression. J Prosthodont.

[7] Thongthammachat S, Moore B, Barco M, Hovijitra S, Brown D, Andres C (2002). Dimensional accuracy of dental casts: influence of tray material, impression material, and time. J Prosthodont.

[8] Gonçalves F, Popoff D, Castro C, Silva G, Magalhães C, Moreira A (2011). Dimensional stability of elastomeric impression materials: a critical review of the literature. Eur J Prosthodont Restor Dent.

[9] Gibelli D, Dolci C, Cappella A, Sforza C (2020). Reliability of optical devices for three-dimensional facial anatomy description: a systematic review and meta-analysis. Int J Oral Maxillofac Surg.

[10] Bohner L, Gamba DD, Hanisch M, Marcio BS, Tortamano Neto P, Laganá Dalva Cruz, Sesma N (2019). Accuracy of digital technologies for the scanning of facial, skeletal, and intraoral tissues: a systematic review. J Prosthet Dent.

[11] Douglas TS (2004). Image processing for craniofacial landmark identification and measurement: a review of photogrammetry and cephalometry. Comput Med Imaging Graph.

[12] Plooij J, Maal T, Haers P, Borstlap W, Kuijpers-Jagtman A, Bergé Stefaan J (2011). Digital three-dimensional image fusion processes for planning and evaluating orthodontics and orthognathic surgery: a systematic review. Int J Oral Maxillofac Surg.

[13] Kook M, Jung S, Park H, Oh H, Ryu S, Cho J, Lee J, Yoon S, Kim M, Shin H (2014). A comparison study of different facial soft tissue analysis methods. J Craniomaxillofac Surg.

[14] Lippold C, Liu X, Wangdo K, Drerup B, Schreiber K, Kirschneck C, Moiseenko T, Danesh G (2014). Facial landmark localization by curvature maps and profile analysis. Head Face Med.

[15] Elbashti M, Sumita Y, Aswehlee A, Seelaus R (2019). Smartphone application as a low-cost alternative for digitizing facial defects: is it accurate enough for clinical application?. Int J Prosthodont.

[16] Camison L, Bykowski M, Lee W, Carlson J, Roosenboom J, Goldstein J, Losee J, Weinberg S (2018). Validation of the Vectra H1 portable three-dimensional photogrammetry system for facial imaging. Int J Oral Maxillofac Surg.

[17] Özsoy Umut, Sekerci R, Hizay A, Yildirim Y, Uysal H (2019). Assessment of reproducibility and reliability of facial expressions using 3D handheld scanner. J Craniomaxillofac Surg.

[18] Cook DA, Erwin PJ, Triola MM (2010). Computerized virtual patients in health professions education: a systematic review and meta-analysis. Acad Med.

[19] Ko B (2018). A brief review of facial emotion recognition based on visual information. Sensors (Basel).

[20] Jeon B, Jeong B, Jee S, Huang Y, Kim Y, Park GH, Kim J, Wufuer M, Jin X, Kim SW, Choi TH (2019). A facial recognition mobile app for patient safety and biometric identification: design, development, and validation. JMIR Mhealth Uhealth.

[21] Burke P, Beard L (1967). Stereophotogrammetry of the face. A preliminary investigation into the accuracy of a simplified system evolved for contour mapping by photography. Am J Orthod Dentofacial Orthop.

[22] Gwilliam JR, Cunningham SJ, Hutton T (2006). Reproducibility of soft tissue landmarks on three-dimensional facial scans. Eur J Orthod.

[23] Kovacs L, Zimmermann A, Brockmann G, Baurecht H, Schwenzer-Zimmerer K, Papadopulos N, Papadopoulos M, Sader R, Biemer E, Zeilhofer H (2006). Accuracy and precision of the three-dimensional assessment of the facial surface using a 3-D laser scanner. IEEE Trans Med Imaging.

[24] Bakirman T, Gumusay MU, Reis HC, Selbesoglu MO, Yosmaoglu S, Yaras MC, Seker DZ, Bayram B (2017). Comparison of low cost 3D structured light scanners for face modeling. Appl Opt.

[25] Ma L, Xu T, Lin J (2009). Validation of a three-dimensional facial scanning system based on structured light techniques. Comput Methods Programs Biomed.

[26] Piccirilli M, Doretto G, Ross A, Adjeroh D (2016). A mobile structured light system for 3D face acquisition. IEEE Sensors J.

[27] Piedra-Cascón Wenceslao, Meyer MJ, Methani MM, Revilla-León Marta (2020). Accuracy (trueness and precision) of a dual-structured light facial scanner and interexaminer reliability. J Prosthet Dent.

[28] Amornvit P, Sanohkan S (2019). The accuracy of digital face scans obtained from 3D Scanners: an in vitro study. Int J Environ Res Public Health.

[29] Knoops PG, Beaumont CA, Borghi A, Rodriguez-Florez N, Breakey RW, Rodgers W, Angullia F, Jeelani NO, Schievano S, Dunaway DJ (2017). Comparison of three-dimensional scanner systems for craniomaxillofacial imaging. J Plast Reconstr Aesthet Surg.

[30] Hassan B, Greven M, Wismeijer D (2017). Integrating 3D facial scanning in a digital workflow to CAD/CAM design and fabricate complete dentures for immediate total mouth rehabilitation. J Adv Prosthodont.

[31] Lo Russo L, Salamini A, Troiano G, Guida L (2020). Digital dentures: a protocol based on intraoral scans. J Prosthet Dent.

[32] Hong S, Noh K (2020). Setting the sagittal condylar inclination on a virtual articulator by using a facial and intraoral scan of the protrusive interocclusal position: a dental technique. J Prosthet Dent.

[33] Revilla-León Marta, Raney L, Piedra-Cascón Wenceslao, Barrington J, Zandinejad A, Özcan Mutlu (2020). Digital workflow for an esthetic rehabilitation using a facial and intraoral scanner and an additive manufactured silicone index: a dental technique. J Prosthet Dent.

[34] Moher D, Liberati A, Tetzlaff J, Altman DG, PRISMA Group (2009). Preferred reporting items for systematic reviews and meta-analyses: the PRISMA statement. PLoS Med.

[35] Whiting PF, Rutjes Anne W S, Westwood Marie E, Mallett Susan, Deeks Jonathan J, Reitsma Johannes B, Leeflang Mariska M G, Sterne Jonathan A C, Bossuyt Patrick M M, QUADAS-2 Group (2011). QUADAS-2: a revised tool for the quality assessment of diagnostic accuracy studies. Ann Intern Med.

[36] Cohen J (1992). Statistical Power Analysis. Curr Dir Psychol Sci.

[37] Higgins JPT, Thompson SG, Deeks JJ, Altman DG (2003). Measuring inconsistency in meta-analyses. BMJ.

[38] DerSimonian R, Kacker R (2007). Random-effects model for meta-analysis of clinical trials: an update. Contemp Clin Trials.

[39] McGuinness LA, Higgins JPT (2020). Risk-of-bias VISualization (robvis): an R package and Shiny web app for visualizing risk-of-bias assessments. Res Synth Methods.

[40] Koban K, Cotofana S, Frank K, Green J, Etzel L, Li Zhouxiao, Giunta Riccardo E, Schenck Thilo L (2019). Precision in 3-dimensional surface imaging of the face: a handheld scanner comparison performed in a cadaveric model. Aesthet Surg J.

[41] Maués C P R, Casagrande M, Almeida R, Almeida M, Carvalho F (2018). Three-dimensional surface models of the facial soft tissues acquired with a low-cost scanner. Int J Oral Maxillofac Surg.

[42] Ten Harkel Timen C, Speksnijder CM, van der Heijden F, Beurskens CHG, Ingels KJAO, Maal TJJ (2017). Depth accuracy of the RealSense F200: low-cost 4D facial imaging. Sci Rep.

[43] Liu C, Artopoulos A (2019). Validation of a low-cost portable 3-dimensional face scanner. Imaging Sci Dent.

[44] Koban KC, Perko P, Etzel L, Li Z, Schenck TL, Giunta RE (2020). Validation of two handheld devices against a non-portable three-dimensional surface scanner and assessment of potential use for intraoperative facial imaging. J Plast Reconstr Aesthet Surg.

[45] Ross MT, Cruz R, Brooks-Richards TL, Hafner LM, Powell SK, Woodruff MA (2018). Comparison of three-dimensional surface scanning techniques for capturing the external ear. Virtual and Physical Prototyping.

[46] Aung S, Ngim R, Lee S (1995). Evaluation of the laser scanner as a surface measuring tool and its accuracy compared with direct facial anthropometric measurements. Br J Plast Surg.

[47] Secher JJ, Darvann TA, Pinholt EM (2017). Accuracy and reproducibility of the DAVID SLS-2 scanner in three-dimensional facial imaging. J Craniomaxillofac Surg.

[48] Ye H, Lv L, Liu Y, Liu Y, Zhou Y (2016). Evaluation of the accuracy, reliability, and reproducibility of two different 3D face-scanning systems. Int J Prosthodont.

[49] Rangel FA, Maal TJ, Bergé Stefaan J, van Vlijmen OJ, Plooij JM, Schutyser F, Kuijpers-Jagtman AM (2008). Integration of digital dental casts in 3-dimensional facial photographs. Am J Orthod Dentofacial Orthop.

[50] Lane C, Harrell W (2008). Completing the 3-dimensional picture. Am J Orthod Dentofacial Orthop.

[51] Yao H, Ge C, Xue J, Zheng N (2017). A high spatial resolution depth sensing method based on binocular structured light. Sensors (Basel).

[52] Sarbolandi H, Lefloch D, Kolb A (2015). Kinect range sensing: Structured-light versus Time-of-Flight Kinect. Computer Vision and Image Understanding.

[53] Jia T, Zhou Z, Gao H (2014). Depth measurement based on infrared coded structured light. Journal of Sensors.

[54] Alfaro-Santafé J, Gómez-Bernal A, Lanuza-Cerzócimo C, Alfaro-Santafé JV, Pérez-Morcillo A, Almenar-Arasanz AJ (2020). Three-axis measurements with a novel system for 3D plantar foot scanning: iPhone X. Footwear Science.

[55] Verhulst A, Hol M, Vreeken R, Becking A, Ulrich D, Maal T (2018). Three-dimensional imaging of the face: a comparison between three different imaging modalities. Aesthet Surg J.

[56] Zaidel DW, Hessamian M (2010). Asymmetry and symmetry in the beauty of human faces. Symmetry.

[57] Terrin N, Schmid CH, Lau J (2005). In an empirical evaluation of the funnel plot, researchers could not visually identify publication bias. J Clin Epidemiol.

[58] Sterne JAC, Sutton AJ, Ioannidis JPA, Terrin N, Jones DR, Lau J, Carpenter J, Rücker Gerta, Harbord RM, Schmid CH, Tetzlaff J, Deeks JJ, Peters J, Macaskill P, Schwarzer G, Duval S, Altman DG, Moher D, Higgins JPT (2011). Recommendations for examining and interpreting funnel plot asymmetry in meta-analyses of randomised controlled trials. BMJ.

