# Supplementary material for: Accuracy of Mobile Device–Compatible 3D Scanners for Facial Digitization: Systematic Review and Meta-Analysis
Source: J Med Internet Res. 2020 Oct 23;22(10):e22228. doi: 10.2196/22228 (PMC7647818; doi:10.2196/22228)
Supplement: Multimedia Appendix 1 [file jmir_v22i10e22228_app1.docx]

**Supplemental table 1.** Commercial mobile-device-compatible face scanners investigated in the studies included.

| **Commercial systems** | **Compatible mobile devices** | **Software/ application** | **Image capture technology** | **Working range** | **Capture speed** | **Processing Speed** | **3D resolution** | **Output format** | **Approximate price (May 28, 2020)** |
| --- | --- | --- | --- | --- | --- | --- | --- | --- | --- |
| Face Camera Pro Bellus (Bellus3D, Campbell, CA, USA) | Android 7.0 with 4GB RAM Windows 8, 10 with 4GB RAM | Bellus3D Face Camera App (Bellus3D) | Dual structured light scanner | 25cm to 60cm (optimal at 30-45cm) | 15-25 sec | SD: Approximately 7 sec (Windows), Approximately 15 sec (Android) HD: Approximately 12 sec (Windows), Approximately 30 sec (Android) | SD Face Model: Approximately 6MB file size (OBJ) with 60,000 triangles and 2K (width) color texture map  HD Face Model: Approximately 24MB file size (OBJ) with 250,000 triangles and 4K (width) color texture map | 3D face model in OBJ with MTL, Color texture map in JPG, 3D face landmarks in OBJ and YML | 500 USD |
| iPhone/ iPad camera (Apple Inc., Cupertino, CA, USA) | Apple IOS devices | Bellus3D Face Application (Bellus3D); Autodesk 123D Catch software (Autodesk, San Francisco, CA, USA); iOS application (tap tap tap LLC, USA) | Photogrammetry/ Infrared structured light | Depend on models | Depend on models | Depend on models | Depend on models | Textured mesh | Depend on models |
| iSense (3D Systems, Rock Hill, SC, USA) | iPhone (iPhone 6, iPhone 6 Plus), iPad (iPad 4, iPad Air, iPad Air 2, iPad mini) | 3DSizeME (v2.0, TechMed3D, Lévis, Qc, Canada) | Infrared structured light | NA | 5-30 sec | Depend on compatible devices | 0.9 (at 0.5 m)– 30 (at 3 m) | Textured mesh | 499 USD |
| Microsoft Kinect (Microsoft, Redmond, WA, USA) | Window notebook | Kinect applications for Microsoft Windows (Microsoft) | Infrared structured light | 50 cm | 30 sec | Depend on compatible devices | VGA resolution (640 × 480 pixels) with 11-bit depth, which provides 2,048 levels of sensitivity | Textured mesh | 250 USD |
| RealSense (Intel, Santa Clara, USA) | Windows, macOS, Android devices | DF_3DScan (Intel, Santa Clara, CA, USA) | Infrared structured light | Approximately 0.5 m | 0.5 ± 0.2 min | 1.2 ± 0.5 min | Depth range: 0.3-1.5 m frame/sec: 30 RGB resolution: 1080p | Textured mesh | 150 USD |
| Scanify (Fuel 3D Technologies, Chinnor, UK) | Windows 7, Windows 8, Mac | Fuel3D Studio software (Fuel 3D Technologies) | Photogrammetry | 10-30 cm | 0.1 sec | Depend on compatible devices | 0.35 mm | STL, PLY, and OBJ | 1500 USD |
| Sense (3D Systems, Rock Hill, SC, USA) | Window notebook, Microsoft Surface™ Pro 2 tablet | Geomagic software (3D Systems) | Infrared structured light | Full 360-degree capture possible | 12.1 ± 6 sec | 1 min (hardware dependent) | 0.76 megapixels | Textured mesh | 399 USD |
| Structure Sensor (Occipital Inc., San Francisco, CA, USA) | iPad (iPad Pro, iPad mini, iPad Air) | Scanner - Structure SDK (Occipital Inc., San Francisco, CA, USA) | Infrared structured light | 0.3 to 5 m | Approximately 20 sec | Depend on compatible devices | 0.13 ± 0.01 polygons/mm^2^ | Textured mesh | 1000 USD |

*Information as provided by the manufactures.
